# Information-Entropy-Based Single Amino Acid Polymorphism Analysis Reveals Functional Variance of Enterovirus 2A Proteases

**DOI:** 10.3390/microorganisms14081616

**Published:** 2026-07-24

**Authors:** Xi Zhu, Zhoule Guo, Qiong Wang, Xing-Yi Ge, Yang Xiao, Ye Qiu

**Affiliations:** 1 Hunan Provincial Key Laboratory of Medical Virology, Hunan Research Center of the Basic Discipline for Cell Signaling, College of Biology, Hunan University, 27 Tianma Rd., Changsha 410012, China; zhuxi@hnu.edu.cn (X.Z.); guozhoule@hnu.edu.cn (Z.G.); xyge@hnu.edu.cn (X.-Y.G.); 2 College of Basic Medicine, Changsha Medical University, Changsha 410219, China; 3 Hunan Provincial Key Laboratory of the Traditional Chinese Medicine Agricultural Biogenomics, Changsha Medical University, Changsha 410219, China

**Keywords:** enterovirus A, 2A protease, single amino acid polymorphism, information entropy, functional diversity

## Abstract

*Enterovirus alphacoxsackie* (EV-A) is a highly diverse viral species containing at least 25 serotypes with diverse biological and clinical characteristics. EV-A can cause diseases ranging from asymptomatic infections to severe neurological disorders, as well as mucocutaneous diseases such as hand, foot, and mouth disease. 2A, a cysteine protease expressed by EV-A, plays critical roles in virus–host interactions. Although 2A orthologs of different EV-A serotypes share consistent protease-catalytic motifs and cleavage patterns, they show functional diversity in interacting with cellular proteins, probably due to distinct protease-independent activities determined by the single amino acid polymorphisms (SAPs) among different 2A orthologs. However, routine sequence alignment and phylogenetic analysis can hardly identify the key SAP sites (kSAPs) contributing to the functional variance, mainly due to the high conservation of the proteins and the unequal weight of SAPs in determining protein function. Herein, we developed Single Amino Acid Polymorphism Statistics (SAAPS), an information-entropy (IE)-based algorithmic pipeline, to identify the functional kSAPs of EV-A 2A. The core principle of the algorithm is that the IE of the kSAPs can be neither too low (highly conserved sites not leading to variance) nor too high (random neutral mutations). Using SAAPS, we identified 56 kSAPs from 2A of 25 EV-A serotypes. Based on the kSAPs, the 2As can be clustered into three major groups with a few outliers, which was distinct from the clustering generated by phylogenetic analysis using the whole amino acid sequences. Functional verification with transcriptomic profiles of HEK-293T cells expressing different 2A variants revealed closer alignment of kSAP clustering than phylogenetic clustering. Notably, EV-A89, an outlier identified by kSAP clustering but not phylogenetic clustering, showed a unique expression pattern with an altered shift in the molecular weight, which suggested that it was related to three SAPs identified by SAAPS. This study presents SAAPS as a useful tool for prioritizing functionally relevant SAPs to guide mechanistic discovery and can be applied to highly conserved proteins like EV-A 2A.

## 1. Introduction

*Enterovirus alphacoxsackie* (EV-A) is a group of non-enveloped, positive-sense, and single-stranded RNA viruses belonging to the *Enterovirus* genus of the *Picornaviridae* family. EV-A can cause diseases ranging from asymptomatic infections to severe neurological disorders such as aseptic meningitis and encephalitis [1,2], as well as mucocutaneous diseases like hand, foot, and mouth disease (HFMD) [3,4,5]. In recent decades, large-scale EV-A outbreaks have frequently been reported, particularly in East and Southeast Asia [6,7,8,9], resulting in a substantial public health burden.

The EV-A genome is approximately 7.4–7.5 kb in length, containing a 5′ untranslated region (UTR), a single open reading frame (ORF), and a 3′ UTR. The ORF encodes a polyprotein precursor that is processed by viral proteases into three segments (P1–P3). The P1 segment encodes four structural proteins (VP1-VP4), whereas the P2 (2A–2C) and P3 (3A–3D) segments encode non-structural proteins [10,11,12]. Based on sequence similarity in the P1 segment and non-structural 2C/3CD regions, the International Committee on Taxonomy of Viruses (ICTV) has classified EV-A into 25 distinct serotypes, including *Coxsackievirus A* (CVA) and other Enterovirus A serotypes [13]. Although these serotypes share conserved genomic features, they exhibit distinct epidemiological and clinical characteristics.

EV-A infection is associated with a broad spectrum of diseases, with clinical outcomes influenced by multiple factors, including viral serotype, biological characteristics, and host immune status. Notable differences exist in the clinical profiles among distinct serotypes. Besides causing typical HFMD, EV-A71, the most extensively studied serotype, frequently leads to severe neurological complications such as brainstem encephalitis, aseptic meningitis, and neurogenic pulmonary edema [14]. In contrast, CVA16, another major causative agent of HFMD [3], usually leads to mild clinical manifestations characterized by faint rash and short-term fever, with rare cases of severe complications [15]. These divergent clinical and biological phenotypes likely reflect serotype-specific differences in viral replication kinetics, polyprotein processing efficiency, and capacity for host immune modulation.

Among the non-structural proteins, the 2A protease plays a critical role in regulating these processes. As a cysteine protease, it cleaves the viral polyprotein to ensure proper assembly of viral particles. Inter-serotype variations in cleavage efficiency potentially contribute to differences in viral replication rates [16]. Moreover, 2A protease modulates host cellular processes by targeting key factors such as the eukaryotic initiation factor eIF4G [17], thereby inhibiting host protein synthesis in favor of viral translation. Additionally, 2A interferes with innate immune pathways, notably the RIG-I/MAVS signaling axis [18], and alters cytoskeletal components [19] to facilitate viral release. Given these pleiotropic roles, 2A protease is regarded as an essential determinant of viral fitness and a promising target for antiviral strategies. Notably, inter-serotype variations in 2A’s protease sequence, catalytic activity, and substrate specificity have been implicated in the observed differences in replication rates, tissue preference, and capacity for immune evasion [16].

In recent years, other serotypes like CVA6 and CVA10 have emerged as significant causative agents of HFMD [4], exhibiting clinical features distinct from those of EV-A71 and CVA16. CVA6 infection often results in atypical HFMD, characterized by a more extensive rash distribution and complex lesion morphology, and is frequently accompanied by onychomadesis and mucosal erosion, suggesting enhanced tropism for skin and mucosal tissues [20]. Besides HFMD, herpangina is also observed in CVA10 infection, characterized by prolonged fever duration in certain cases and clinical manifestations that may mimic herpesvirus infections [21], requiring differential diagnosis. The emergence of these serotypes with diverse clinical presentations highlights the importance of serotype-specific factors.

As with many RNA viruses, EV-A exhibits a high mutation rate, driven by the lack of proofreading activity in its RNA-dependent RNA polymerase [22,23]. Genetic variability arises predominantly through point mutations and frequent recombination events, contributing to antigenic drift, immune escape, and enhanced adaptability [24,25,26]. However, traditional sequence analyses often treat all observed mutations equally without distinguishing between neutral, functionally irrelevant polymorphisms and mutations that may impact viral phenotypes. This limitation highlights the need for refined analytical strategies capable of identifying functionally relevant variations. In practice, the study of single amino acid polymorphisms (SAPs) [27,28] facilitates the identification of non-synonymous mutations related to alteration of protein function. SAPs have been employed in various virological analyses to elucidate determinants of virulence, receptor usage, and immune evasion [29,30,31]. However, a key challenge in current SAP analyses lies in distinguishing functional variations from the background of neutral mutations.

To address the limitations above, we developed Single Amino Acid Polymorphism Statistics (SAAPS), an information-entropy (IE)-based algorithmic pipeline, to identify the functional kSAPs of EV-A 2A. The core principle of the algorithm is that the IE of the kSAPs can be neither too low (highly conserved sites not leading to variance) nor too high (random neutral mutations). Using EV-A 2A proteases from 25 serotypes as a proof-of-concept dataset, we demonstrate that SAAPS-driven clustering can identify functional associations that are not evident from conventional phylogenetics and can guide experimental validation. This work positions SAAPS as a useful tool for prioritizing functionally relevant SAPs, guiding mechanistic discovery, and can be applied to highly conserved proteins like EV-A 2A.

## 2. Materials and Methods

### 2.1. Data Collection and Sequence Processing

Information on EV-A serotypes was obtained from the Picornaviridae database (https://picornaviridae.com/), and the amino acid sequences of 2A proteases were obtained from the National Center for Biotechnology Information (NCBI) (https://www.ncbi.nlm.nih.gov/). A total of 25 EV-A serotypes were selected for analysis to ensure comprehensive representation of genetic diversity. Sequences containing ambiguous characters, low-quality regions, or incomplete coding regions were filtered out to maintain high data integrity. Any sequence with unidentified codons or incorrect formatting was replaced with higher-quality alternatives from the database. All the viral sequences used in this study are listed in Supplementary Table S1. These sequences correspond to prototype strains for each EV-A serotype. While a single representative sequence does not capture natural intra-serotype variation, it provides a consistent benchmark for method comparison.

### 2.2. Sequence Analysis and Phylogenetic Classification

To investigate the evolutionary relationships among EV-A serotypes, multiple sequence alignment was performed using MAFFT [32], followed by sequence identity analysis using BioAider [33]. The phylogenetic trees were constructed using Maximum Likelihood (ML) and Neighbor-Joining (NJ) methods. Evolutionary distances were estimated using substitution models selected via model-fitting analyses performed with ProtTest [34] and MEGA 7 [35] (the model results predicted can be found in Supplementary Tables S2 and S3). The robustness of tree topologies was assessed using bootstrap resampling to ensure reliable clustering of viral strains. Comparative analysis between traditional phylogenetic approaches and SAP-based classification was conducted to evaluate the efficacy of SAP clustering in delineating viral subgroups.

### 2.3. SAAPS Workflow Used in This Study

Single Amino Acid Polymorphism Statistics (SAAPS) is a Python- and R-based workflow developed to analyze polymorphic amino acid sites in aligned protein sequences. In general, SAAPS can identify single amino acid polymorphisms (SAPs), calculate site-level Shannon entropy and information content (IC), filter polymorphic sites by user-defined IC ranges, convert selected residues into numerical features, and perform dimensionality-reduction and clustering analyses. General software functions and command-line usage are described in the Supplementary SAAPS Manual and at https://github.com/xiaosheep01/SAAPS (accessed on 25 August 2025).

In this study, we used a defined SAAPS workflow for EV-A 2A proteases. The workflow consisted of sequence alignment, SAP identification, entropy and IC calculation, IC-based kSAP selection, AAindex1 encoding, PCA dimensionality reduction, and OPTICS clustering. This section describes only the implementation used for the present EV-A 2A analysis.

### 2.4. Entropy, Information Content, and kSAP Selection

In this study, the identification of kSAPs in the EV-A 2A protease was performed using Shannon information entropy and SAAPS. Shannon entropy, a concept originating from information theory, quantifies the uncertainty or diversity within a system. In this study, we employed “Information Content” (IC) as a metric to assess the level of polymorphism at each site. For each aligned position j, the frequency of amino acid a was denoted as $p_{j,a}$. Gaps and ambiguous residues were excluded. The number of observed amino acid states at position j was denoted as $K_{j}$, and the number of possible amino acid states used for IC calculation was denoted as N. Because only the 20 standard amino acids were considered, N = 20.

Shannon entropy at position j was calculated as:

(1) $$H_{j}=-\sum_{a=1}^{K_{j}}\;p_{j,a}\;\log_{2}(p_{j,a})$$

Information content was calculated as:

(2) $$IC(j)={log}_{2}\left(N\right)-H_{j}$$

Under this definition, a conserved site has an entropy close to 0 and IC close to $\log_{2}(20)\;=\;4.32193$, whereas a highly variable site has a higher entropy and lower IC. Thus, a higher IC indicates greater conservation, while a lower IC indicates greater amino acid variability.

To select kSAPs, positions were grouped by $K_{j}$. The mean IC was calculated for each $K_{j}$ group. The upper cutoff was derived from the mean IC of sites with two observed amino acid states, with a 5% operational tolerance, yielding 4.022. The lower cutoff was derived from the mean IC values of sites with four and five observed amino acid states, with a 5% operational tolerance, yielding 2.735. This gave an empirical candidate interval of 2.735–4.022.

To validate the biological relevance of this interval, we performed a sensitivity analysis by testing multiple IC ranges (from 1.677 to 4.322 to 3.274–4.322) and examined their clustering patterns against an external biological feature—the known host ranges of the serotypes (non-human primate, baboon, human). This information was used only for post hoc assessment, not for threshold derivation. Only the 2.735–4.022 range produced clustering that was consistent with the host range information (see Section 3.2), thereby confirming its operational suitability for this EV-A 2A dataset. This interval is dataset-specific and should not be interpreted as a universal threshold for other proteins or sequence collections. For other datasets, users are encouraged to perform a similar sensitivity analysis.

### 2.5. AAindex Encoding, PCA, and OPTICS Clustering

Residues at the selected kSAP positions were encoded using the Amino Acid Index (AAindex) [36]. Features were selected from the AAindex1 database (release 20170213), which contains descriptors of amino acid physicochemical properties. All 566 AAindex1 physicochemical descriptors were initially considered. Descriptors containing missing values for the 20 standard amino acids were removed, leaving 553 descriptors. Each residue at each selected kSAP position was therefore represented by a 553-dimensional vector.

For each EV-A serotype, AAindex vectors from the selected kSAP positions were concatenated in alignment order. Because 56 kSAP positions were selected, each serotype was represented by 56 × 553 = 30,968 features. The final matrix contained 25 rows, corresponding to the 25 EV-A serotypes, and 30,968 columns, corresponding to the concatenated AAindex features. AAindex feature values were standardized before PCA.

PCA was used as a deterministic linear dimensionality-reduction step to summarize the dominant variation in the high-dimensional AAindex matrix. The first two principal components explained 61.55% of the total variance (PC1, 42.71%; PC2, 18.84%) and were used for two-dimensional visualization and exploratory clustering. OPTICS was then applied to PC1 and PC2 using Euclidean distance, min_samples = 2, max_eps = 30, cluster_method = “dbscan”, and min_cluster_size = 2. Under these settings, PCA and OPTICS were deterministic and did not require random initialization or random seeds.

This PCA-OPTICS analysis was used as a proof-of-concept application of SAAPS to EV-A 2A proteases. It was not intended as a comprehensive benchmark of all possible dimensionality-reduction and clustering methods. Therefore, the resulting clusters were interpreted as candidate functional groupings supported by kSAP patterns and transcriptomic comparisons rather than as a definitive or universally optimal classification.

### 2.6. RNA-Seq Library Preparation, Sequencing, and Data Analysis

To validate the clustering results of polymorphic sites and assess their association with functional phenotypes, bulk RNA-seq was performed on HEK-293T cells expressing the 2A proteases from all 25 EV-A serotypes. Two independent biological replicates were prepared for each serotype, along with an empty vector control. Cells were harvested 24 h post-transfection, and total RNA was extracted using TRIzol reagent (15596026, Invitrogen, Shanghai, China), followed by DNase I treatment. RNA quality was determined by examining A260/A280 with a Nanodrop OneC spectrophotometer (Thermo Fisher Scientific, Shanghai, China). RNA integrity was confirmed by 1.5% agarose gel electrophoresis. Qualified RNAs were finally quantified by Qubit3.0 with the Qubit RNA Broad Range Assay kit (Q10210, Life Technologies, Shanghai, China).

A total of 2 μg of RNA was used for stranded Bulk RNA sequencing library preparation using the KC-Digital Stranded mRNA Library Prep Kit for Illumina (DR08502, Wuhan Seqhealth, Wuhan, China) following the manufacturer’s instructions. This kit uses a unique molecular identifier (UMI) of 8 random bases to label the pre-amplified cDNA molecules, enabling removal of PCR duplication bias. The library products corresponding to 200–500 bp were enriched, quantified, and finally sequenced on a DNBSEQ-T7 sequencer (MGI Tech, Shenzhen, China) with the PE150 model.

Raw reads were filtered with Trimmomatic (version 0.36) to remove low-quality reads and reads contaminated with adaptor sequences. Clean reads were first clustered according to the UMI sequences, in which reads with the same UMI sequence were grouped into the same cluster. Reads in the same cluster were compared to each other by pairwise alignment, and then reads with a sequence identity over 95% were extracted to a new sub-cluster. After all sub-clusters were generated, multiple sequence alignment was performed to achieve one consensus sequence for each sub-cluster. After these steps, any errors and biases introduced by PCR amplification or sequencing were eliminated.

The de-duplicated consensus sequences were used for standard RNA-seq analysis. They were mapped to the reference genome of homo sapiens (GRCh38_release110) using STAR software (version 2.5.3a) with default parameters. Gene-level read counts were quantified with featureCounts (Subread-1.5.1), and RPKM values were calculated. Genes differentially expressed between groups were identified using the edgeR package (version 3.12.1). A gene was considered significantly differentially expressed if it satisfied |log_2_FC| > 1 and a false discovery rate (FDR) < 0.05.

Gene Ontology (GO) functional enrichment and Kyoto Encyclopedia of Genes and Genomes (KEGG) pathway enrichment analyses were conducted utilizing the R package clusterProfiler [37,38]. Subsequently, for each identified cluster, intersection analyses of KEGG pathways and DEGs were carried out to determine key pathways or genes commonly shared among different clusters.

### 2.7. Cell Culture

HEK-293T (National Collection of Authenticated Cell Cultures, Shanghai, China) cells were cultured in Dulbecco’s modified Eagle’s medium (DMEM; C11995500BT, Gibco, Shanghai, China), supplemented with 10% fetal bovine serum (A5256701, Gibco, Shanghai, China) and 1% penicillin–streptomycin (SL6040, Coolaber, Beijing, China). All cells were cultured at 37 °C in a humidified incubator with 5% CO_2_.

### 2.8. Plasmids and Transfection

All plasmids used in this study were commercially synthesized by Tsingke Biotechnology Co., Ltd. (Beijing, China) and verified by Sanger sequencing. For plasmid transfection, HEK-293T cells were seeded in 12-well plates (70% confluence) one day before transfection. On the day of transfection, the cell culture medium was changed, and cells were transfected with the indicated plasmids (1000 ng/well) using Lipofectamine LTX and PLUS Transfection Reagent (Invitrogen, Shanghai, China) according to the manufacturer’s instructions.

### 2.9. Western Blotting

At 24 h post-transfection, cells were washed with phosphate-buffered saline (PBS) and lysed with RIPA buffer (AWB0138a, Abiowell, Changsha, China) supplemented with 1% protease inhibitor cocktail and phosphatase inhibitor cocktail (78441, Thermo Fisher Scientific, Shanghai, China. The lysates were incubated on ice for 30 min and centrifuged at 12,000× *g* for 15 min at 4 °C. Supernatants were boiled with 6× loading buffer at 98 °C for 10 min. Proteins were resolved on SDS-PAGE gels and transferred to polyvinylidene difluoride (PVDF) membranes (IPVH00010, Merck, Shanghai, China) that had been pre-activated with methanol. The PVDF membranes were then blocked with 5% skim milk for 1 h, followed by incubation with primary antibody overnight at 4 °C. After three washes with 1× TBST (G0004-1L, Servicebio, Wuhan, China), the membranes were incubated with secondary antibody for 1 h at room temperature. The membranes were then washed three times with 1× TBST, and protein bands were visualized using an ECL substrate (K1232, APExBIO, Houston, TX, USA) and a chemiluminescence imaging system (OI 600MF, Bio-OI, Guangzhou, China). Mouse monoclonal anti-Myc antibody (M20002S 19C2, Abmart, Shanghai, China) was used at a 1:2000 dilution in 1× TBST. HRP-conjugated beta actin monoclonal antibody (AN00295HP, Elabscience, Wuhan, China) was used at a 1:2000 dilution in 1× TBST. HRP-conjugated goat anti-mouse IgG secondary antibody (A21010) was purchased from Abbkine and used at a 1:5000 dilution in 1× TBST.

## 3. Results

### 3.1. Design of Single Amino Acid Polymorphism Statistics (SAAPS)

**Figure 1:**
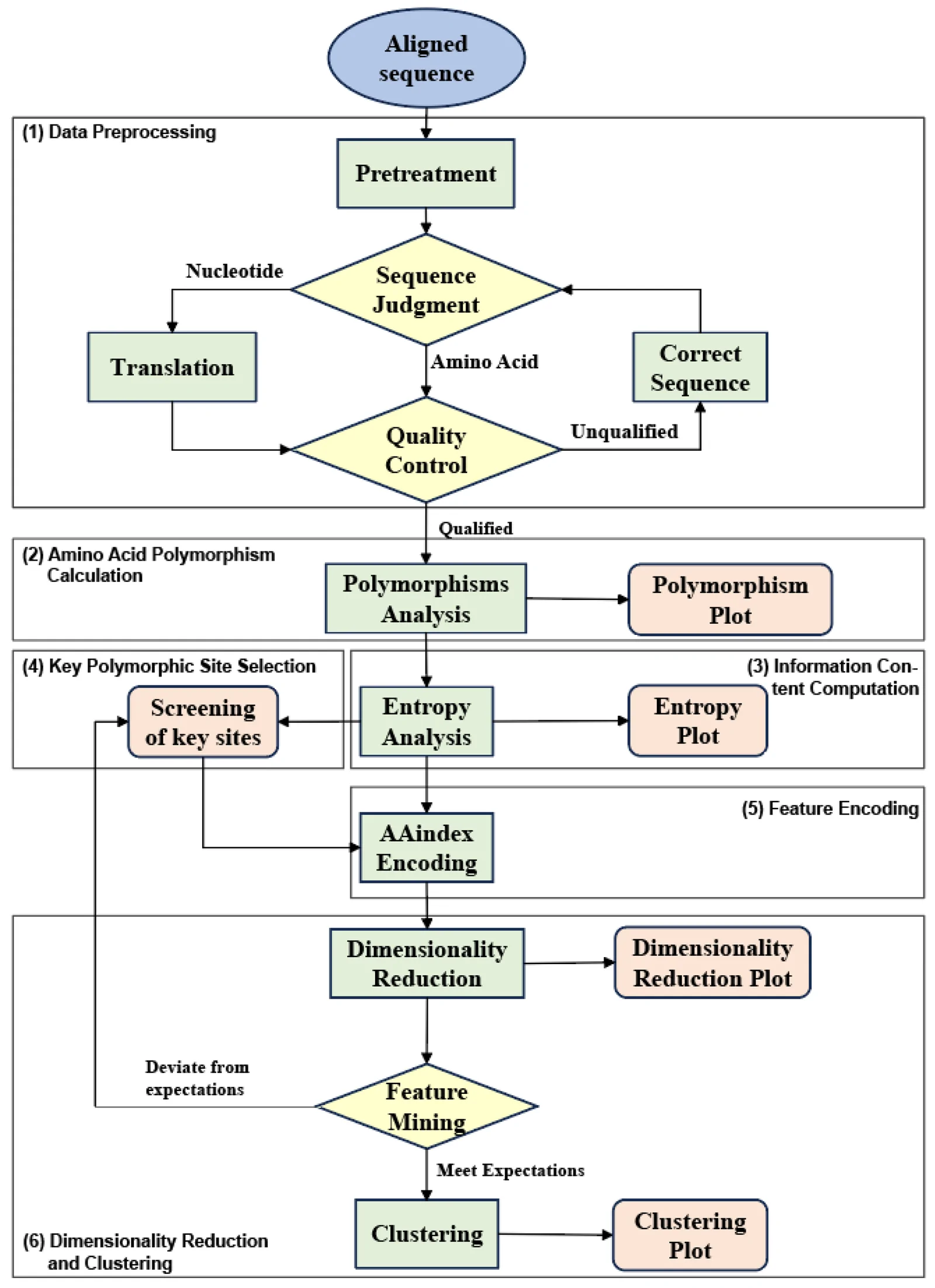
Overview of the SAAPS analysis pipeline. The SAAPS (Single Amino Acid Polymorphism Statistics) pipeline is built with Python 3.10, integrating multiple functional modules for the analysis of amino acid sequences. The workflow consists of six main modules: (1) Data Preprocessing; (2) Amino Acid Polymorphism Calculation; (3) Information Content Computation; (4) Key Polymorphic Site Selection; (5) Feature Encoding; (6) Dimensionality Reduction and Clustering. Color coding: blue, input; green, processing; yellow, decision; orange, output.

SAAPS is an analytical pipeline built with Python 3.10, integrating multiple functional modules. The overall workflow is illustrated in Figure 1 and comprises the following key steps.

1. Data Preprocessing: This initial step involves input validation, sequence type identification and conversion, and quality control. If preprocessed data or intermediate results are provided, this step can be skipped.
2. Amino Acid Polymorphism Calculation: A site is defined as polymorphic if at least two amino acid variants are present. Parameters can be adjusted to generate polymorphism distribution plots.
3. Information Content Computation: Based on Shannon entropy, the information content of each site is calculated, and a corresponding distribution plot is generated.
4. Key Polymorphic Site Selection: The default threshold is determined by the minimum and maximum values computed from the input sequences, though users can customize this range. If no threshold is set, all sites are included.
5. Feature Encoding: SAAPS supports one-hot encoding and AAindex transformation, converting amino acid residues into numerical representations for statistical analysis.
6. Dimensionality Reduction and Clustering: Given the high-dimensional nature of polymorphic site encoding, dimensionality reduction is applied to simplify data complexity while preserving key features. Subsequently, clustering algorithms are used to classify the reduced dataset.

SAAPS is designed for multi-sequence analysis and is not limited to enteroviruses, making it applicable to a wide range of sequence data.

### 3.2. Evolutionary Relationships and IC Threshold Determination for Clustering

**Figure 2:**
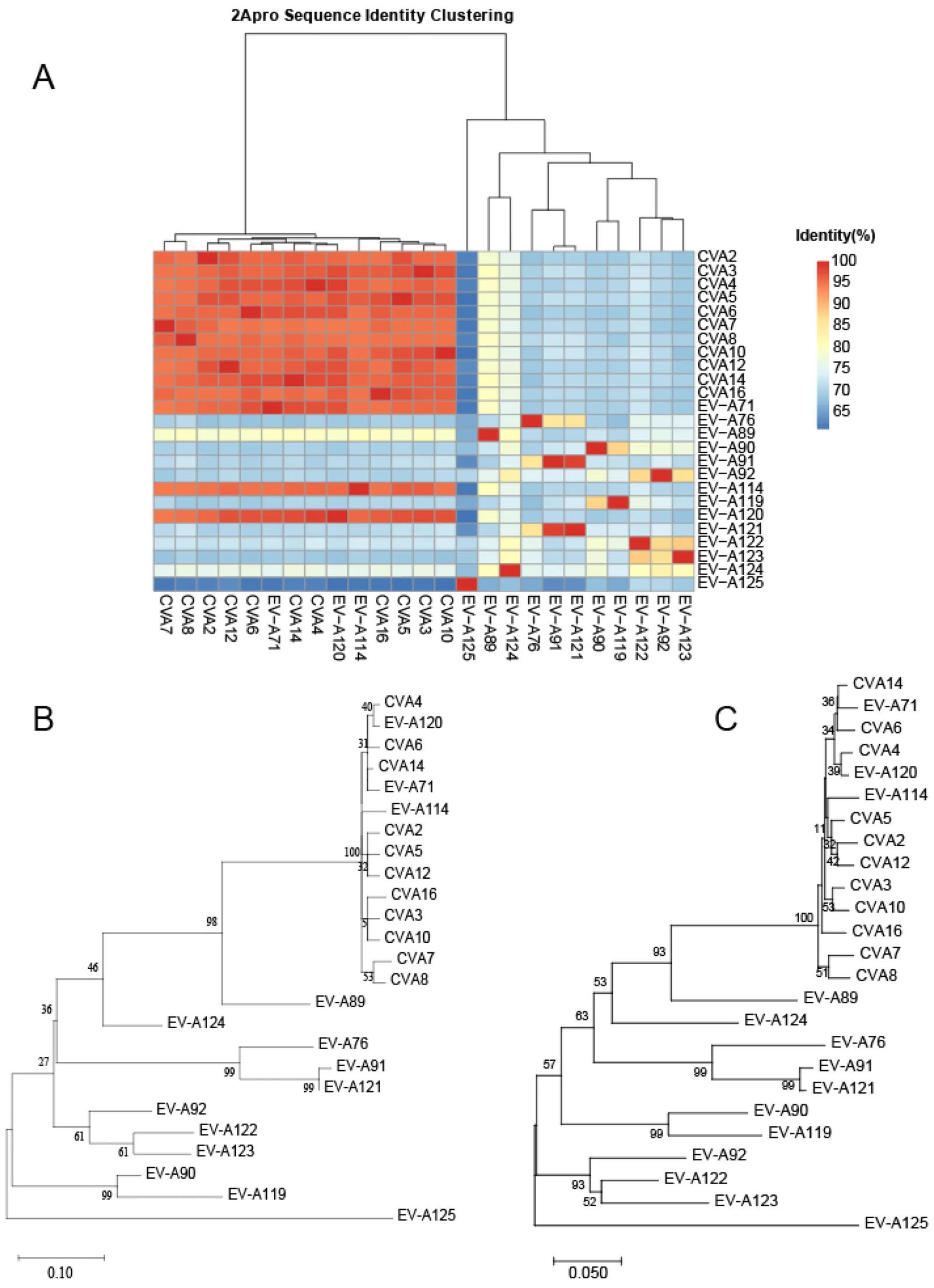
Evolutionary relationships and clusters of EV-A 2A proteases. (**A**) Sequence identity heatmap of the 2A protease among 25 EV-A serotypes. Pairwise sequence identities were calculated using BioAider and visualized using the R package ggplot2. The color gradient ranges from blue (low similarity) to red (high similarity). Hierarchical clustering dendrograms are displayed above the matrix. (**B**) Neighbor-Joining (NJ) phylogenetic tree of the 25 EV-A serotypes based on 2A protease amino acid sequences. The tree was constructed using the Poisson model with bootstrap values (500 replicates) shown at the nodes. The scale bar represents 0.10 substitutions per site. (**C**) Maximum Likelihood (ML) phylogenetic tree of the 25 EV-A serotypes based on 2A protease amino acid sequences. The tree was constructed using the LG + G substitution model (selected by ProtTest and MEGA) with bootstrap values shown at the nodes. The scale bar represents the number of substitutions per site.

Figure 2A presents the sequence conservation heatmap of 25 EV-A strains, which was generated using BioAider and visualized in R. CVA2, CVA3, CVA4, CVA5, CVA6, CVA7, CVA8, CVA10, CVA12, CVA14, CVA16, EV-A71, EV-A114, and EV-A120 exhibit high average pairwise sequence identity (>96.23%) and form a distinct cluster. Other EV-A strains display weaker clustering, though EV-A76, EV-A91, and EV-A121 (90% average pairwise identity), and EV-A92, EV-A122, and EV-A123 (87.56% average pairwise identity) show closer relationships. EV-A124 and EV-A125 are more divergent, with EV-A125 exhibiting the lowest average pairwise sequence identity (64.57%).

Phylogenetic trees of EV-A 2A protease, constructed via NJ and ML (Figure 2B,C), display similar topologies, consistent with the sequence conservation heatmap. The best fit substitution models selected by ProtTest and MEGA are provided in Supplementary Tables S2 and S3, respectively. Most human EV-A, including CVA2~CVA16 and EV-A71/114/120, form a typical clade, in which the bootstrap values of most branches are low, indicating that the phylogenetic analysis performed poorly on highly conserved EV-A 2As. Beyond this clade, the phylogenetic relations of the less conserved 2As are relatively clear and consistent with phenotypic features such as the viral host range, which were used to determine the best IC threshold for functional clustering using SAAPS.

**Figure 3:**
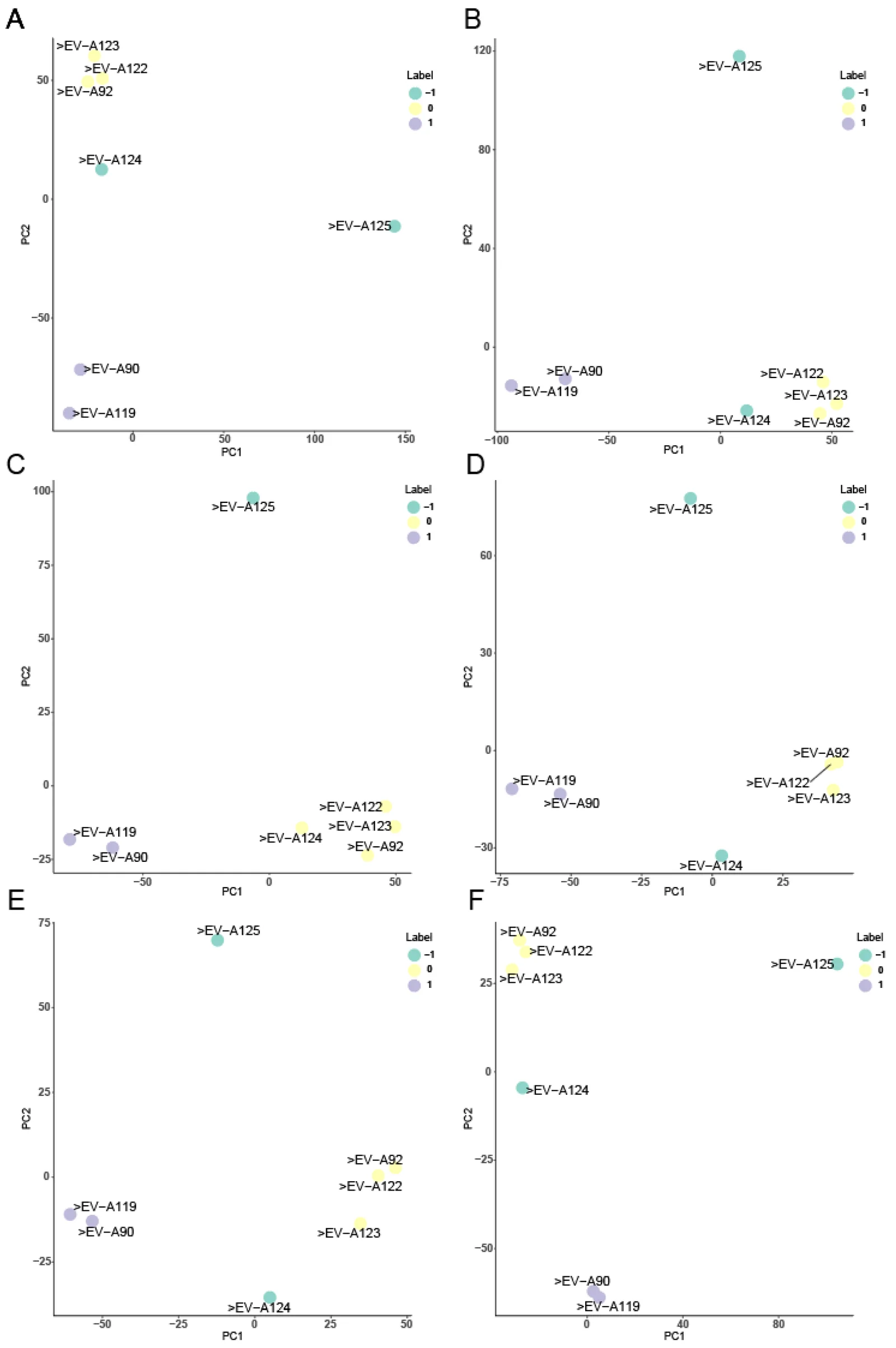
Effect of IC threshold selection on clustering of kSAPs. PCA clustering results of kSAPs under six different IC thresholds: (**A**) 1.677–4.322, (**B**) 1.761–4.022, (**C**) 2.735–4.022, (**D**) 3.022–4.022, (**E**) 3.274–4.022, and (**F**) 3.274–4.322.

The clustering topology of less conserved 2As was evaluated with multiple IC ranges, including 1.677–4.322, 1.761–4.022, 2.735–4.022, 3.022–4.022, 3.274–4.022, and 3.274–4.322 (Figure 3A–F, respectively). To determine the best IC range for 2A clustering, we then examined their clustering patterns against an external feature—the known host ranges of the serotypes. Under all IC ranges, EV-A90 and EV-A119 consistently formed a distinct pair. Likewise, EV-A92, EV-A122, and EV-A123 always clustered together, and EV-A125 remained an outlier in every range. However, the clustering position of EV-A124 varied with the threshold: under broader IC ranges (e.g., Figure 3A,B,D–F), EV-A124 appeared as an outlier similar to EV-A125; only within the range 2.735–4.022 (Figure 3C) did EV-A124 cluster together with EV-A92, EV-A122, and EV-A123. Notably, this grouping pattern is consistent with the host range information: EV-A92, EV-A122, EV-A123, and EV-A124 are all known to infect non-human primates (*Macaca* spp.), whereas EV-A125 is known to infect baboons. This consistency provides supportive evidence for the operational choice of the 2.735–4.022 threshold, but does not imply a direct causal link between 2A sequence variations and host tropism. Therefore, the 2.735–4.022 range was selected as the operational IC range for all subsequent analyses. The following sections will characterize the key SAP sites (kSAPs) and further validate the clustering through transcriptomic profiling.

### 3.3. Identification and Selection of Polymorphic Sites in EV-A 2A Protease

**Figure 4:**
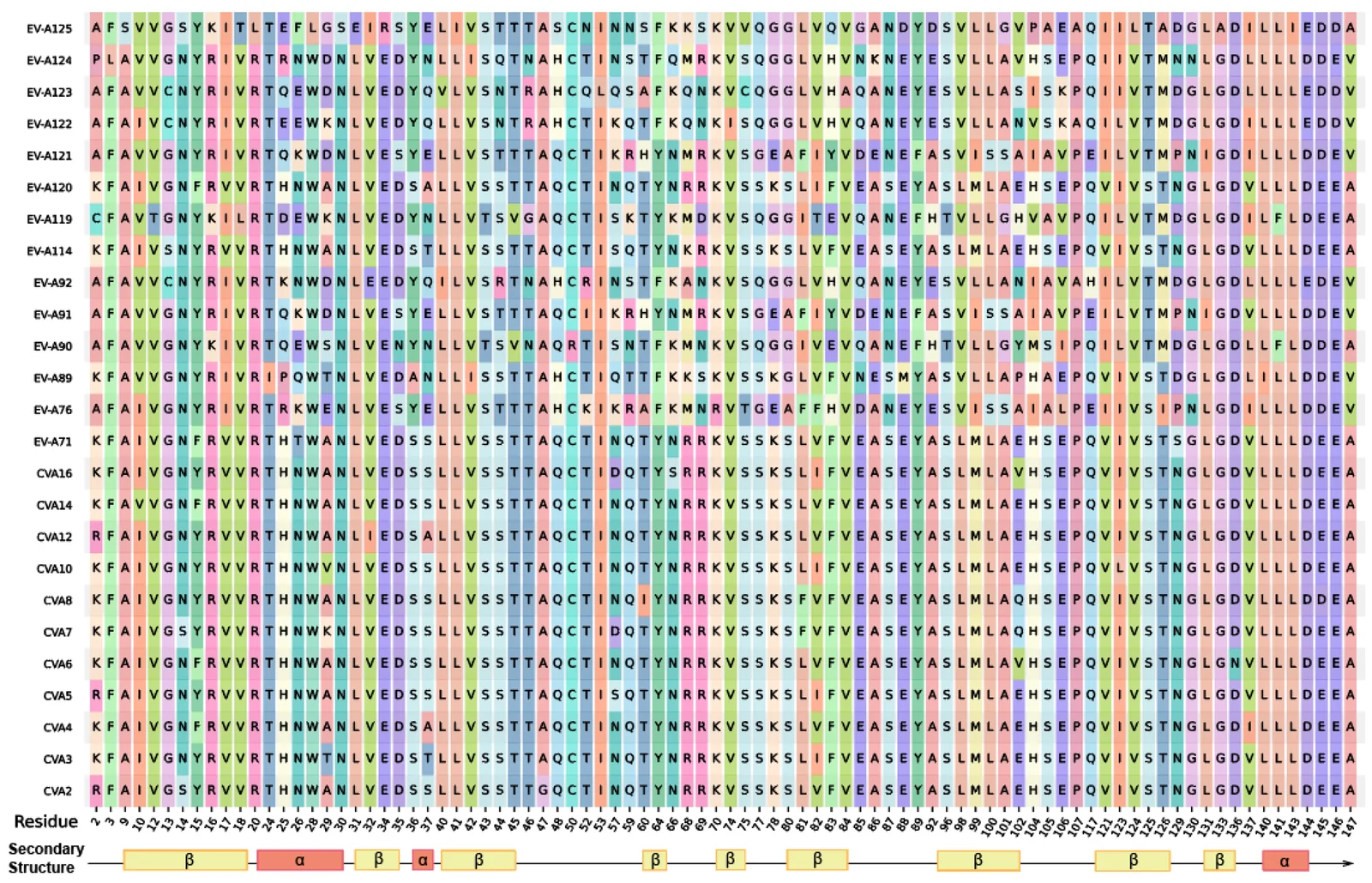
Distribution of polymorphic sites in the 2A protease. Distribution of polymorphic sites across the 2A protease sequence. A total of 88 polymorphic sites were identified; amino acid types are distinguished by unique colors. Secondary structure elements are annotated below the sequence, with α-helices highlighted in red and β-strands highlighted in yellow.

**Figure 5:**
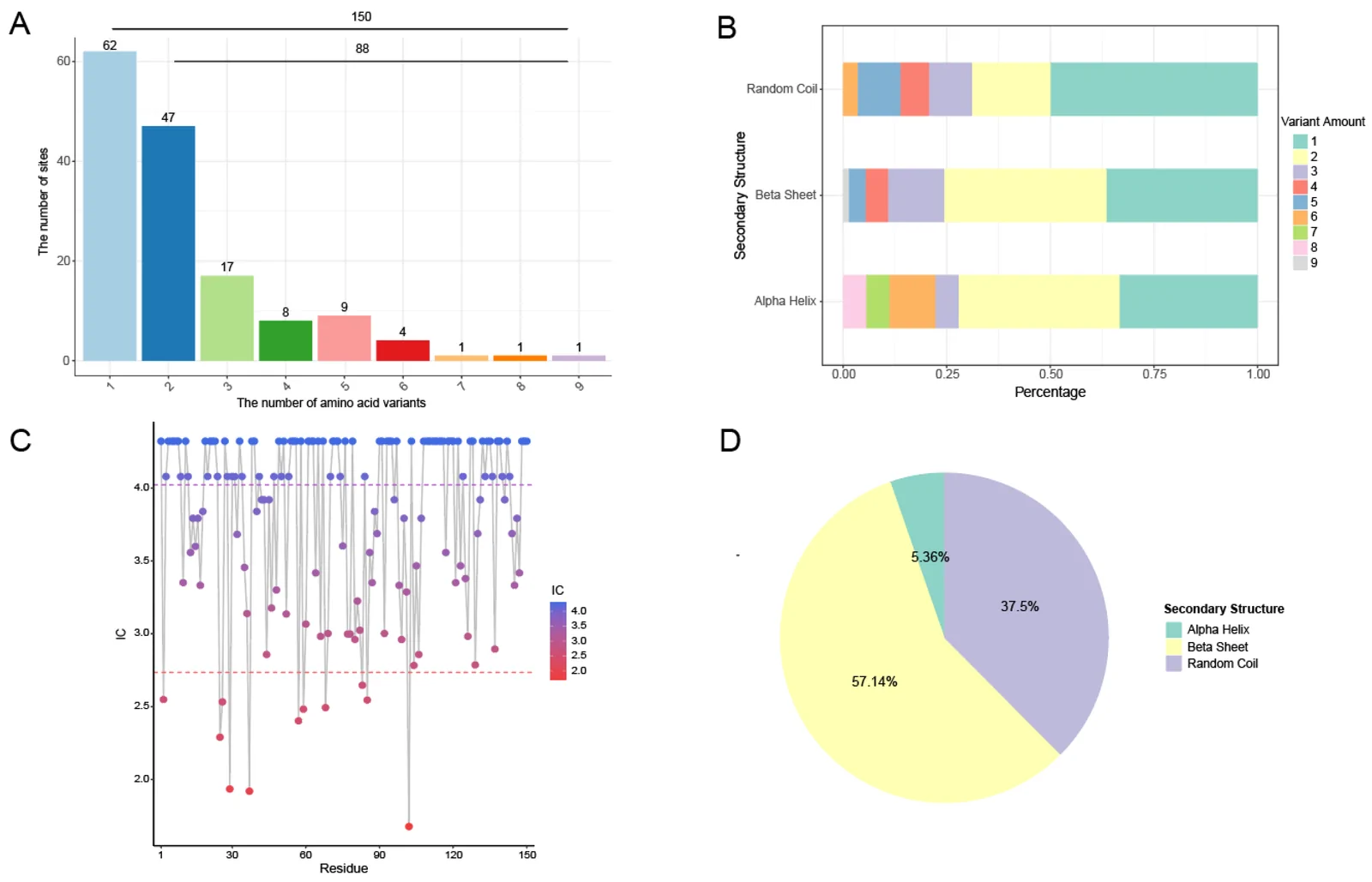
Polymorphism and information content analyses of the EV-A 2A protease. (**A**) Distribution of polymorphic sites by variant number. Among 150 total positions, 88 were polymorphic (≥2 variants). (**B**) Secondary structure distribution of polymorphic sites. For each secondary structure type, the percentages of sites with 1–9 variants are shown. (**C**) IC values for each site. Dashed lines indicate the selected threshold range (2.735–4.022). (**D**) Secondary structure distribution of the 56 kSAPs. The percentages of sites located in α-helices, β-strands, and random coils are presented.

Polymorphism analysis of the 2A protease in 25 EV-A strains was conducted using SAAPS, and the identified polymorphic sites are shown in Figure 4. The EV-A 2A protease consists of 150 amino acids. A total of 88 sites exhibited polymorphism, accounting for 58.67% (n = 88/150) of all sites (Figure 5A), while non-polymorphic sites constituted 41.33% (n = 62/150). Among these, position 102 displayed the highest variability, with nine amino acid variants. The majority of polymorphic sites were relatively stable, with 47 sites containing only two amino acid variants.

A comparison with the secondary structure (Figure 5B) revealed that sites with higher amino acid variability (6–8 variants) were primarily located within α-helices, comprising 22.11% of all α-helical positions. In contrast, less variable sites (two to four variants) were predominantly distributed in both α-helices (44.45%) and β-strands (58.11%). Sites with five variants (n = 9) were found across all secondary structure types (α-helices, β-strands, and random coils). Notably, position 102, which contains nine variants (the highest observed variability), is located within a β-strand, indicating that hypervariable sites are not exclusively confined to α-helices.

Shannon entropy was used to calculate the Information Content (IC) for each site (Figure 5C). The IC values ranged from 1.677 to 4.322. To ensure comprehensive inclusion of polymorphic sites while excluding highly variable sites or non-polymorphic positions, and based on host-guided functional clustering, a threshold range of 2.735–4.022 was established. In total, 56 polymorphic sites fell within this range and were classified as potential kSAPs (Supplementary Table S4 provides the IC values for all 150 positions). Among these 56 kSAPs (Figure 5D), 5.36% were located within α-helices, while 57.14% were found in β-strands, suggesting structural relevance in their functional variability.

### 3.4. Dimensionality Reduction and Clustering Analysis of kSAPs

**Figure 6:**
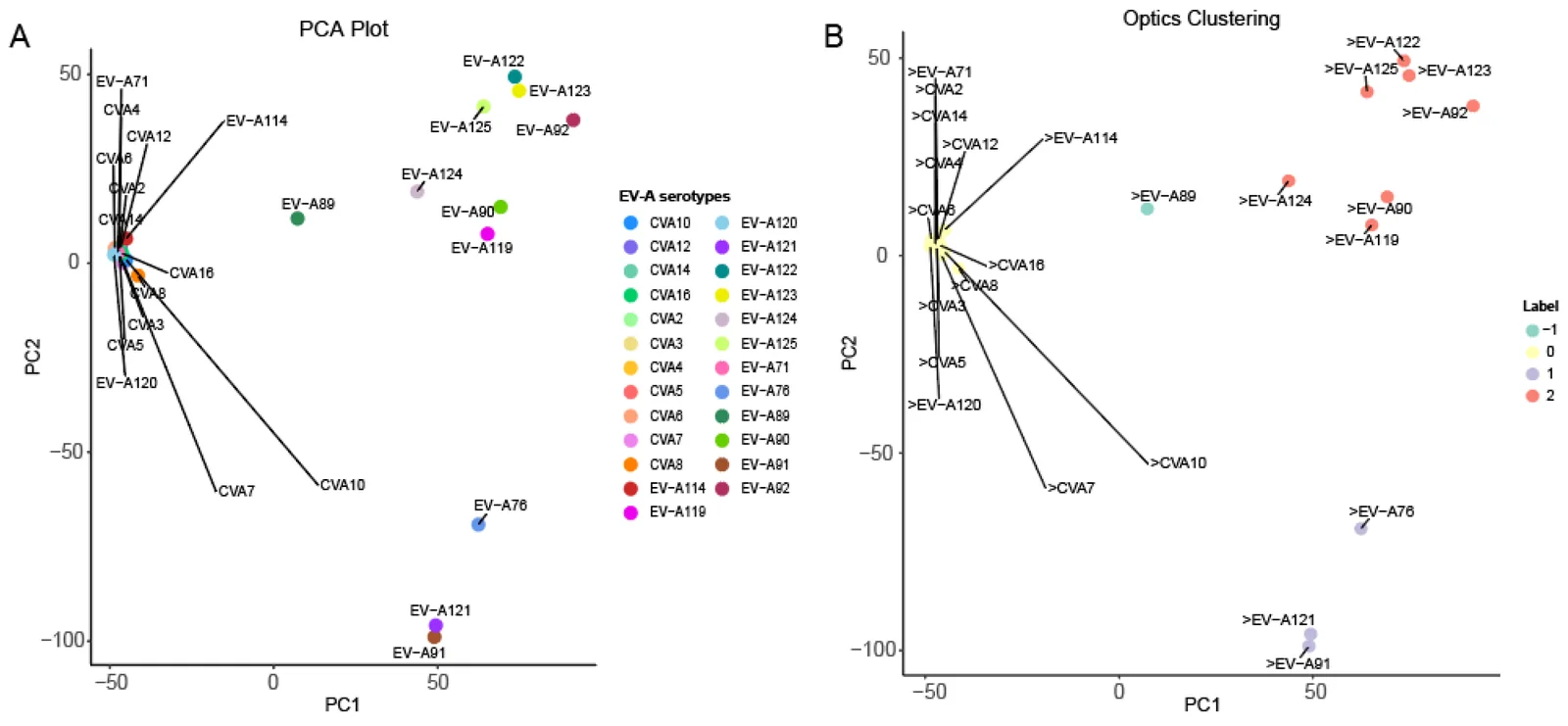
Dimensionality reduction and clustering analyses of kSAPs. (**A**) Principal Component Analysis (PCA) of the selected polymorphic sites revealed three major clusters. (**B**) OPTICS clustering identified EV-A89 as a noise point, aligning with its phylogenetic position. In the OPTICS clustering color legend, “−1”, ”0”, “1” and “2” represent noise point, group 1, group 2, and group 3, respectively.

PCA was applied to the selected polymorphic sites, revealing three major clusters (Figure 6A). The leftmost cluster, containing CVA2, CVA3, CVA4, CVA5, CVA6, CVA7, CVA8, CVA10, CVA12, CVA14, CVA16, EV-A71, EV-A114, and EV-A120, aligned with the phylogenetic tree. However, the upper-right cluster (EV-A90, EV-A92, EV-A119, EV-A122, EV-A123, EV-A124, EV-A125) and the lower-right cluster (EV-A76, EV-A91, EV-A121) showed discrepancies. Notably, within the lower-right cluster, EV-A76 was more distant from EV-A91 and EV-A121, indicating greater divergence.

OPTICS clustering (Figure 6B) confirmed the PCA results, identifying EV-A89 as a noise point, consistent with its phylogenetic position. Unlike the phylogenetic tree, EV-A90, EV-A92, EV-A119, EV-A122, EV-A123, EV-A124, and EV-A125 formed a distinct group, suggesting shared characteristics.

### 3.5. Functional Validation of SAAPS Clustering by Transcriptomics

**Figure 7:**
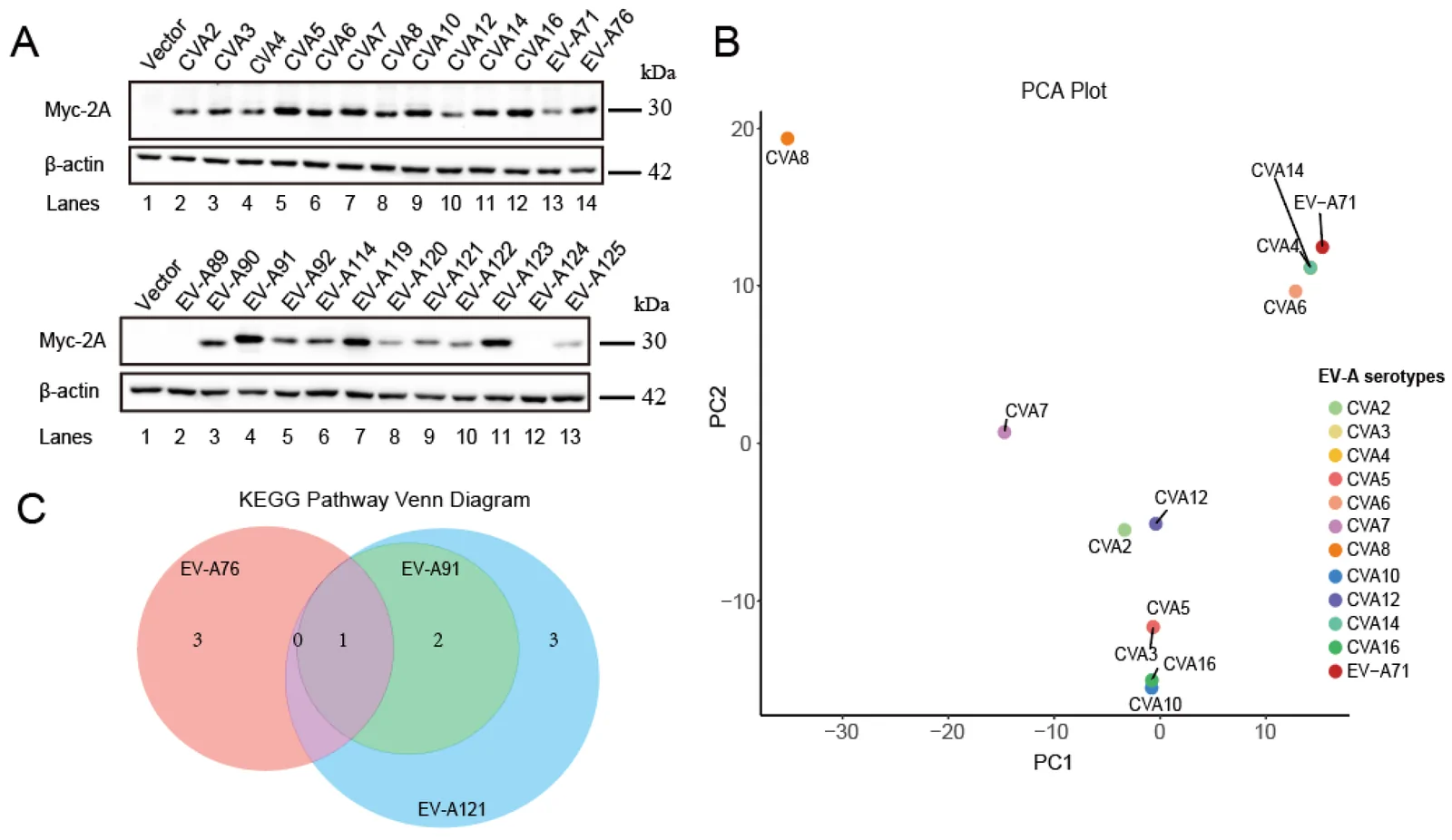
Transcriptomic phenotypic analyses of EV-A 2A protease. (**A**) Western blot of HEK-293T cells expressing 2A protease from 25 EV-A serotypes. Empty vector was used as a negative control. Top membrane (lanes 1–14): vector, CVA2-CVA8, CVA10, CVA12, CVA14, CVA16, EV-A71, and EV-A76. Bottom membrane (lanes 1–13): vector, EV-A89, EV-A90, EV-A91, EV-A92, EV-A114, EV-A119, EV-A120, EV-A121, EV-A122, EV-A123, EV-A124, and EV-A125. The expected molecular weights of EV-A 2A (30 kDa) and β-actin (42 kDa) are indicated on the right. Representative of three independent experiments. (**B**) PCA clustering of 12 serotypes from Group 1 (CVA2–CVA8, CVA10, CVA12, CVA14, CVA16, and EV-A71). (**C**) Venn diagram of shared KEGG pathways among EV-A76, EV-A91, and EV-A121.

As proof-of-concept, we expressed 25 EV-A 2A variants in HEK-293T cells and performed transcriptome sequencing analysis to test whether SAAPS-based clustering aligns better with functional phenotypes than phylogenetic groups. Western blot showed the expression of all 25 2A proteins (Figure 7A).

To better resolve the internal structure of the densely packed leftmost cluster in Figure 5, we performed an independent re-clustering analysis on 12 serotypes (Figure 7B). Phylogenetic analysis (Figure 2C) showed that CVA3 and CVA10, as well as CVA14 and EV-A71, were, respectively, clustered into single clades, whereas our clustering analysis (Figure 7B) revealed closer topological relationships between CVA3 and CVA5, and between CVA14 and CVA4. Transcriptome data further supported this finding: the number of shared differentially expressed genes between CVA3 and CVA5, and between CVA14 and CVA4 (Supplementary Table S5), was higher than that of their corresponding phylogenetic counterparts, providing suggestive evidence. Supplementary Table S5 provides the full list of common DEG counts for each OPTICS cluster group. Notably, the two methods remained highly consistent in the classification of most serotypes (the clustering of CVA2 and CVA12). This suggests that, in this dataset, information entropy-based clustering does not contradict phylogenetic relationships. Instead, it can serve as a complementary approach that captures functional associations not evident from conventional phylogenetics. These observations provide hypothesis-generating cross-omics support for understanding the phenotypic diversity of the 2A protease. Furthermore, EV-A76’s divergence from EV-A91 and EV-A121 may be attributed to functional pathway differences. As illustrated in the Venn diagram (Figure 7C), EV-A121 shares KEGG pathways with EV-A91, whereas EV-A76 and EV-A121 have only one common pathway, supporting their greater evolutionary distance.

To further assess whether the chosen IC threshold (2.735–4.022) captures biologically meaningful groupings, we examined the transcriptomic data independently of the IC-based clustering. The transcriptomic data provided independent support for the IC threshold selection. To further examine whether the observed shift in clustering under the 2.735~4.022 threshold reflected underlying biological similarity, common DEGs were analyzed for EV-A124 in comparison with EV-A92, EV-A122, and EV-A123 (Supplementary Table S6). Supplementary Table S6 shows the common DEG counts among EV-A92, EV-A122, EV-A123, and EV-A124 under different IC thresholds. Furthermore, when EV-A124 was classified as an outlier under broader IC ranges, a total of 30 DEGs were found to be shared among the four viruses. However, within the 2.735~4.022 threshold where EV-A124 clustered with Group 2 viruses, 28 common DEGs remained. The high degree of overlap suggests that EV-A124 exhibits transcriptomic characteristics similar to other Group 2 serotypes, supporting its inclusion within the cluster under this threshold.

### 3.6. EV-A89 as an Outlier: Unusual Expression Behavior of Its 2A Protease

**Figure 8:**
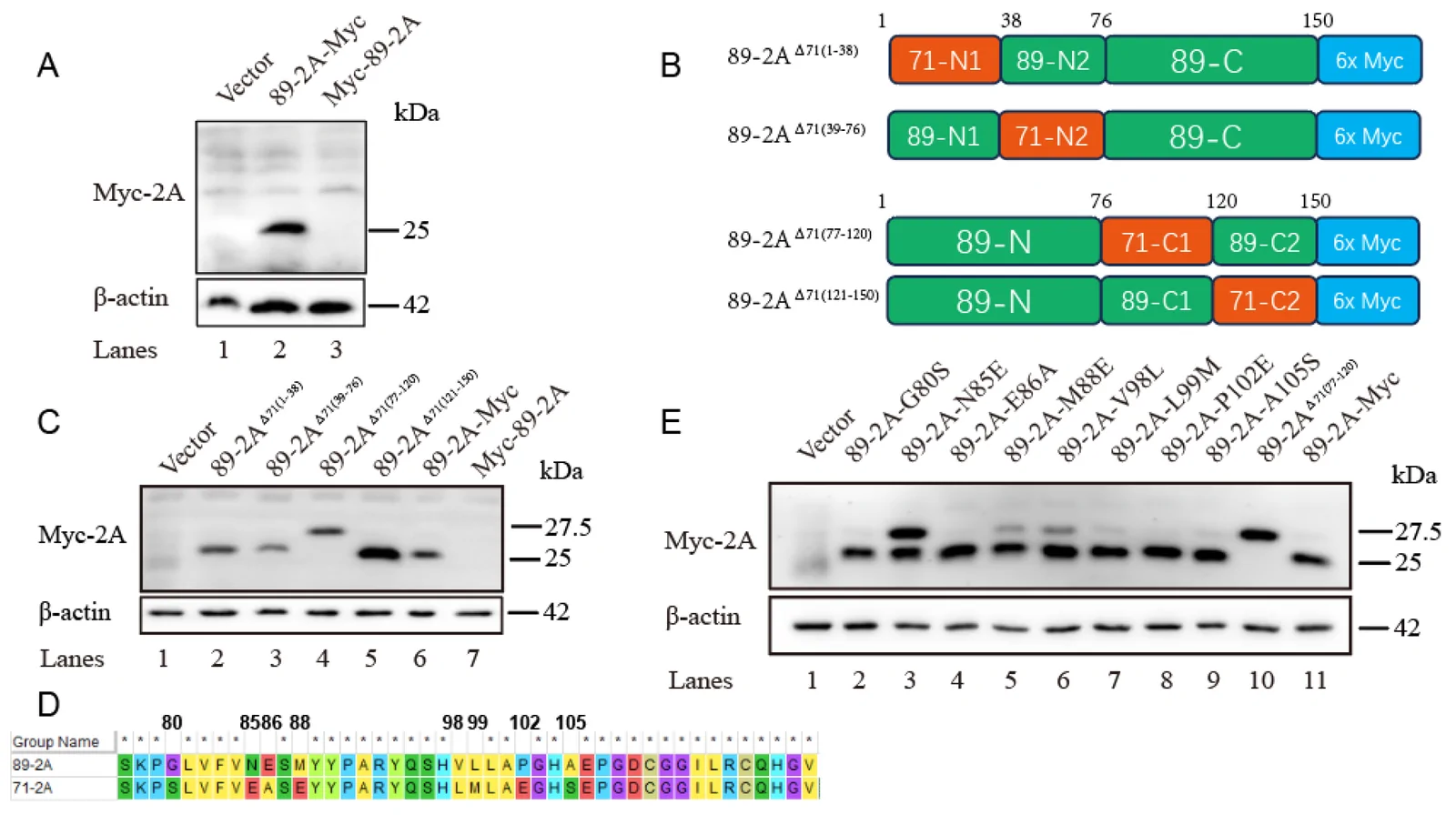
Experimental verification of the kSAPs responsible for the unique expression pattern of EV-A89 2A. (**A**) Western blot of HEK-293T cells transfected with empty vector, C-terminal Myc-tagged EV-A89 2A (89-2A-Myc), or N-terminal Myc-tagged EV-A89 2A (Myc-89-2A). Lanes 1–3: vector, 89-2A-Myc, and Myc-89-2A. The positions of the 89-2A-Myc band (~25 kDa) and β-actin (42 kDa) are indicated on the right. (**B**) Schematic representation of four 2A fusion chimeras: 89-2A^Δ71(1–38)^, 89-2A^Δ71(39–76)^, 89-2A^Δ71(77–120)^, and 89-2A^Δ71(121–150)^. Amino acid boundaries (1, 38, 76, 120, 150) are indicated above the diagrams. All constructs carry a C-terminal 6×Myc tag. (**C**) Western blot of HEK-293T cells expressing the four fusion chimeras and controls. Lanes 1–7: vector, 89-2A^Δ71(1–38)^, 89-2A^Δ71(39–76)^, 89-2A^Δ71(77–120)^, 89-2A^Δ71(121–150)^, 89-2A-Myc, and Myc-89-2A. The 89-2A-Myc control migrates at ~25 kDa. Replacement of residues 77–120 shifts the band to ~27.5 kDa (lane 4), whereas the other chimeras retain the ~25 kDa band. Observed molecular weights are indicated on the right. (**D**) Amino acid sequence alignment of EV-A71 2A and EV-A89 2A within the region spanning residues 77–120. The positions of different residues 80, 85, 86, 88, 98, 99, 102, and 105 are marked above the alignment. The asterisks above the sequences indicates the identical residues. (**E**) Western blot of HEK-293T cells expressing eight point mutants of EV-A89 2A and controls. Lanes: 1, vector; 2–9, point mutants (80, 85, 86, 88, 98, 99, 102, 105); 10, 89-2A^Δ71(77–120)^; 11, 89-2A-Myc. Observed molecular weights are indicated on the right of the blot. All Western blot analyses were performed three times independently; representative blots are shown.

To test whether SAAPS could pinpoint a serotype with unusual properties, we examined EV-A89, an outlier identified from previous PCA results. To elucidate this observation, we investigated the expression behavior of its 2A protease. Initially, we believed that the 2A band of EV-A89 2A was simply weaker than the other EV-A 2A. However, during individual expression studies, we often failed to detect a band near 30 kDa. This led us to hypothesize that EV-A89 2A protease might undergo unusual post-translational processing, resulting in removal of the N-terminal 6x-Myc tag and consequent loss of detection. Therefore, we repositioned the 6x-Myc tag from the N-terminus to the C-terminus, which revealed a target band (25 kDa) smaller than expected (30 kDa), consistent with an altered processing event (Figure 8A).

To map the region responsible for this altered shift in the molecular weight, we selected EV-A71 2A (which shows normal expression) for domain-swap experiments. Based on the secondary structure of the 2A protease, we constructed four 2A fusion chimeras: 89-2A^Δ71(1–38)^, 89-2A^Δ71(39–76)^, 89-2A^Δ71(77–120)^, and 89-2A^Δ71(121–150)^ (Figure 8B). Specifically, in 89-2A^Δ71(1–38)^, the N-terminal residues 1–38 of EV-A89 2A were replaced by the corresponding region of EV-A71 2A, yielding EV-A71(1–38)-EV-A89(39–150). In 89-2A^Δ71(39–76)^, residues 39–76 of EV-A89 2A were substituted with EV-A71 residues 39–76, producing EV-A89(1–38)-EV-A71(39–76)-EV-A89(77–150). The remaining two constructs (89-2A^Δ71(77–120)^ and 89-2A^Δ71(121–150)^) involved analogous replacements in the middle (77–120) and C-terminal (121–150) regions, respectively. All constructs carried a C-terminal 6×Myc tag. The Western blot result (Figure 8C) revealed that replacement of the middle segment (residues 77–120) shifted the band from ~25 kDa to ~27.5 kDa, which is still slightly smaller than the expected 30 kDa. The other chimeras (1–38, 39–76, and 121–150) retained the ~25 kDa band. This indicates that the 77–120 region contributes to the altered migration.

To further identify critical residues, we aligned the amino acid sequences of EV-A71 and EV-A89 2A within the 77–120 region and identified eight polymorphic positions (Figure 8D). Site-directed mutagenesis targeting each of these eight residues was performed. Overexpression of the point mutants revealed that mutations at residues 85, 88, and 98 each produced two protein bands: one corresponding to 89-2A-Myc (~25 kDa) and another co-migrating with the 89-2A^Δ71(77–120)^ chimera (~27.5 kDa) (Figure 8E). This suggests that these residues contribute to the altered processing behavior, although single mutations do not completely abolish the phenomenon. Notably, all three residues are located within kSAPs identified by our SAAPS analysis, exhibiting 5, 3, and 2 amino acid variants among EV-A serotypes, respectively.

KEGG and GO pathway enrichment analyses revealed that EV-A89 uniquely possessed the KEGG pathway “Pathways in cancer” (hsa05200) and GO term “Ossification” (GO:0001503). Notably, *PTGS2*, an inflammation-related gene, was identified within this pathway, potentially explaining EV-A89’s classification as an outlier.

## 4. Discussion

The main contribution of this study is the development of SAAPS, an information-entropy-based pipeline that prioritizes polymorphic sites likely under functional selection. As a proof-of-concept, we applied SAAPS to EV-A 2A proteases. Traditional phylogenetic analyses, typically based on whole-genome or conserved region similarity, often fail to capture serotype-specific functional differences. In contrast, SAAPS-derived clustering showed closer alignment with transcriptomic phenotypes and uncovered a serotype (EV-A89) with an altered expression pattern.

Entropy-based filtering distinguishes functional polymorphisms from background noise. Of the 88 polymorphic sites identified in EV-A 2A protease, 56 (63.6%) met our IC threshold criteria (2.735–4.022). This filtering strategy is grounded in evolutionary theory: sites with an IC < 2.735 (low entropy, near-complete conservation) likely represent structurally or catalytically essential residues where variation is strongly deleterious, consistent with the invariant catalytic triad (His-Asp-Cys) in picornaviral 2A proteases [39,40,41]. Conversely, sites with an IC > 4.022 (high entropy, hypervariable) may reflect neutral drift or lineage-specific adaptations without functional consequences. The intermediate IC range thus captures sites under diversifying selection; precisely, the variants most likely to contribute to phenotypic diversity. The threshold boundaries were empirically derived from the dataset distribution, balancing the risk of splitting biological groups against merging distinct lineages. The lower bound (IC = 2.735) corresponds to sites with exactly two amino acid variants at near-equal frequencies (*p* ≈ 0.5), representing the simplest case of functional polymorphism. The upper bound (IC = 4.022) approximates the entropy of four equally likely variants, beyond which additional diversity provides diminishing functional information. Notably, only the 2.735–4.022 range consistently classified EV-A124 with Group 2 viruses while maintaining EV-A125 as an outlier. This clustering pattern was consistent with both the transcriptomic profiles and, independently, with the known host ranges of these serotypes, where EV-A92/122/123/124 are non-human primate isolates, and EV-A125 is baboon-derived. These consistencies provide supportive evidence for the operational choice of this threshold, but do not imply a direct causal link between 2A polymorphisms and host tropism. Therefore, this threshold can be interpreted as a suitable operational threshold for this EV-A 2A dataset, but it should not be presented as a universally optimal threshold. For other proteins, other viruses, or expanded sets of EV-A sequences, the IC distribution should be recalculated and a similar threshold sensitivity analysis performed.

The divergence between phylogenetic groupings and SAAPS-based clustering arises because conventional phylogeny treats all positions equally, while SAAPS focuses on sites under diversifying selection (IC 2.735–4.022). This explains why, for example, EV-A90, EV-A92, and EV-A119 cluster together in SAAPS but not in the phylogenetic tree. A limitation of this study is that only one representative sequence per serotype was analyzed. This choice was made to ensure standardized and reproducible comparisons between SAAPS and conventional phylogeny, as the primary goal was to introduce and benchmark the SAAPS pipeline rather than to perform a comprehensive serotype-level evolutionary analysis. Nevertheless, we recognize that intra-serotype variation could affect the clustering results, and future work with multiple isolates per serotype will be needed to assess the robustness of our SAAPS-based groupings.

Using SAAPS for systematic identification, 88 polymorphic sites were detected within the 2A protease, of which 56 were classified as key SAP sites (kSAPs) based on information entropy metrics. These critical polymorphic residues exhibited a non-random distribution. Among the 56 key sites, only 5.36% resided in α-helices, whereas 57.14% were located in β-strands. This pronounced bias suggests the possibility that β-sheet structures accommodate functional variation more readily than α-helices in the 2A protease, potentially reflecting the catalytic architecture where strand flexibility modulates substrate access without compromising core stability [16,42]. As the 2A protease is known to orchestrate viral polyprotein processing, suppress host translation, and modulate innate immunity, mutations at these positions could represent candidate determinants that influence viral replication efficiency and immune evasion, although direct evidence is needed.

Classification of EV-A strains based on these filtered polymorphic sites revealed distinct groupings not captured by conventional phylogenetic trees. Strains such as EV-A90, EV-A92, EV-A119, and EV-A122 to EV-A125, which exhibited ambiguous relationships in genome-wide analyses, clustered coherently. Similarly, EV-A124 was reclassified into Group 2 (EV-A92, EV-A122, EV-A123), supported by 28 shared DEGs. Notably, CVA3 and CVA5 share 223 common DEGs—substantially higher than the 46 shared between phylogenetically closer CVA3/CVA10. Likewise, CVA14/CVA4 clustering is supported by 55 common DEGs, compared to only 21 between CVA14 and EV-A71, as predicted by traditional methods.

The outlier status of EV-A89 is particularly significant: although it belongs to the SCARB2-dependent receptor group containing neurovirulent EV-A71 and CVA7 [43,44], transcriptomic data indicate that it uniquely activates PTGS2-mediated inflammatory pathways, potentially offering a hypothesis to explain this outlier observation. Furthermore, we observed that EV-A89 2A exhibits an unusual expression behavior, producing a band of approximately 25 kDa instead of the expected 30 kDa observed for other EV-A 2As. Through domain-swap mapping, we found that replacement of the middle segment (residues 77–120) shifted the band from ~25 kDa to ~27.5 kDa, whereas the other chimeras retained the ~25 kDa band. This indicates that the 77–120 region contributes to the altered shift in the molecular weight. Within this region, mutational analysis identified three residues (85, 88, and 98) that contribute to the altered processing behavior. Each mutant produced two protein bands: one corresponding to 89-2A-Myc (~25 kDa) and another co-migrating with the 89-2A^Δ71(77–120)^ chimera (~27.5 kDa). Notably, these three residues are located within kSAPs identified by our SAAPS analysis. However, the exact molecular basis of the altered migration remains unknown. We acknowledge that additional experiments would be required to distinguish between possible mechanisms (e.g., protease-dead mutant control, degradation, altered post-translational modification). Further investigation is needed to determine the underlying mechanism.

These findings underscore the distinctive capability of SAAPS in resolving functional relevance: by selectively analyzing information entropy-filtered polymorphisms, SAAPS captures phenotypic associations undetectable by whole-genome phylogenetics. Hence, SAAPS could potentially provide a refined analytical framework for molecular epidemiology and outbreak risk assessment, although this remains a future application requiring validation with independent datasets and live virus models. These observations challenge the reliance on genome-wide sequence similarity alone for viral classification, highlighting the necessity of integrating evolutionary selection signals and protein functional domain information into taxonomic frameworks. The clustering results suggest that subtle, functionally relevant polymorphisms—rather than overall genetic identity—may generate hypotheses for future studies on viral phenotypes.

Moreover, while structural proteins have historically dominated enterovirus phylogenetic and antigenic studies [45,46], the centrality of non-structural proteins, particularly proteases like 2A, is increasingly apparent. The functional impact of polymorphisms within non-structural regions, especially those involved in host interaction and immune suppression, warrants closer attention in understanding viral adaptability and functional alteration. Importantly, the entropy-based selection of polymorphic sites demonstrated here refines clustering stability and biological relevance, correcting misclassifications introduced by indiscriminately incorporating all sequence variations. This approach not only enhances viral classification accuracy but also offers practical avenues for further research. Identifying serotype-specific or group-specific polymorphisms within functionally critical regions such as 2A may inform the design of broad-spectrum antiviral agents targeting conserved functional sites. Additionally, integrating polymorphism-based clustering with transcriptomic profiling provides a basis for phenotype-driven viral surveillance, offering the potential to generate hypotheses for future studies.

In this study, we expressed individual 2A proteases in HEK-293T cells to profile host transcriptomic alterations. HEK-293T cells are widely used for such studies due to their high transfection efficiency, robust protein expression, and well-characterized background, which together allow clear detection of 2A-mediated effects. It should be emphasized that this transcriptomic analysis is exploratory, with only two biological replicates per condition and no formal batch-effect correction; it serves as a hypothesis-generating screen rather than a definitive phenotype benchmark. While this approach successfully eliminates confounding effects from other viral proteins and enables direct attribution of observed changes to 2A sequence variations, it does not fully represent the complexity of viral infection. Whole-virus infection involves multiple steps (attachment, entry, replication, assembly) and the concerted action of many viral factors, including structural proteins and other non-structural proteins. Therefore, the transcriptomic signatures identified here should be interpreted as candidate functional differences that are hypothesis-generating and require validation in the context of whole-virus infection. Future work using reverse genetics or direct comparison with infection by authentic EV-A serotypes will be essential to confirm the in vivo relevance of our findings.

Taken together, these findings provide new insights into the molecular determinants of functional diversity of EV-A 2A proteases. By selectively analyzing evolutionarily relevant polymorphisms in the 2A protease, this study establishes a more biologically coherent classification framework and highlights the need to reassess traditional sequence-based methods when addressing functional and clinical diversity within closely related viral species.

## 5. Conclusions

In summary, we developed SAAPS, a novel algorithmic pipeline for SAP analysis, based on the principle that SAPs related to functional diversity of homologous proteins tend to contain a moderate level of information entropy (variance). Verified with transcriptomic alterations caused by ectopic expression of EV-A 2A, SAAPS showed closer alignment with transcriptomic phenotypes than whole-sequence-based methods in this proof-of-concept dataset for functional clustering of the proteins and for kSAP screening. Therefore, these findings suggest that SAAPS can be a useful tool for sequence-function analysis on highly conserved proteins, and that information entropy is a relevant factor to consider in the analysis of protein variance.

## Data Availability

The raw RNA-seq data generated in this study have been deposited in the NCBI Sequence Read Archive (SRA) under BioProject accession numbers PRJNA1466219, PRJNA1466221, PRJNA1466223, and PRJNA1466225. All other data supporting the findings of this study are contained within the article and its Supplementary Materials.

## Supplementary Materials

The following supporting information can be downloaded at: https://www.mdpi.com/article/10.3390/microorganisms14081616/s1, Table S1: All virus sequences used in the study; Table S2: Results of EV-A 2A protease protest detection; Table S3: Results of EV-A 2A protease MEGA detection; Table S4: Results of IC values for all sites of 2A protease; Table S5: The number of common DEGs in each group; Table S6: Changes in common DEGs among EV-A92, EV-A122, EV-A123, and EV-A124. Supplementary SAAPS Manual.

## Abbreviations

The following abbreviations are used in this manuscript:

EV-A	*Enterovirus alphacoxsackie*
CVA	*Coxsackievirus A*
SAPs	Single amino acid polymorphisms
SAAPS	Single Amino Acid Polymorphism Statistics
kSAPs	Key SAP sites
HFMD	Hand, foot, and mouth disease
ORF	Open reading frame
ML	Maximum Likelihood
NJ	Neighbor-Joining
PCA	Principal Component Analysis
IC	Information Content
OPTICS	Ordering Points to Identify the Clustering Structure
DEGs	Differentially expressed genes
KEGG	Kyoto Encyclopedia of Genes and Genomes
GO	Gene Ontology
ICTV	The International Committee on Taxonomy of Viruses
NCBI	The National Center for Biotechnology Information
