# Supplementary material for: Information-Entropy-Based Single Amino Acid Polymorphism Analysis Reveals Functional Variance of Enterovirus 2A Proteases
Source: Microorganisms. 2026 Jul 24;14(8):1616. doi: 10.3390/microorganisms14081616 (PMC13515151; doi:10.3390/microorganisms14081616)
Supplement: Supplementary file 1 [file microorganisms-14-01616-s001.zip › Supplementary SAAPS Manual.pdf]

SAAPS

Single Amino Acids Polymorphism Statistics (SAAPS) is a Python3-based character interface program that can be used and deployed on multiple systems, including Windows, Linux, and MacOS. The software is mainly used to quickly calculate single amino acid polymorphism (SAP), Shannon information entropy at each site of the sequence, and to analyze sequence features and cluster analysis by using Onehot encoding and dimensionality reduction algorithms.

The raw input file of the SAAPS software is very simple, as long as the sequence file after alignment (nucleotide or amino acid), it can be used for processing and analysis. The main workflow of SAAPS is as follows: Firstly, by analyzing the characteristics of each sequence, the SAPs and Shannon information entropy of each point are calculated, and the corresponding result figures can be drawn at the same time. Then SAAPS screens the key sites according to the specified information entropy range, and transforms them into quantificable data by using Onehot encoding. Finally, the certain dimensionality reduction algorithm (PCA or t-SNE) is used to calculate the similarity level between sequences and draw the clustering scatter plot.

1. Download and install

SAAPS is developed via Python 3, and you can get and install in following ways.

1.1 Pip method

SAAPS has been distributed to the standard library pf PYPI, and can be easily installed by the tool pip.

1.2 Local installation

In addition to the pip method, you can download and install it using the file setup.py.

2. Getting help

You can use "-h" or "--help" to get the help document.

3. Examples

This chapter uses the test data ('testSeq.txt' in the test folder) provided by the software as an example to describe the main functions of SAAPS. Note that all sample files in this manual are generated by the software using test data (**"** testSeq.txt **"**), and users can follow the tutorial to learn it before exploring other features on their own.

3.1 Preparation for raw materials

Users need to prepare an alignment sequence file (nucleotide or amino acid file), the alignment process can be completed by MAFFT and other software, and the format of the sequence file after alignment should be "sequence name + (line/multiple lines) sequence body" structure.

3.2 Sequence preprocessing

Before the subsequent analysis, it is usually necessary to format the sequence, such as whether the nucleotide sequence needs to be translated into amino acids, whether the sequence name needs to be changed, and so on. This allows us to get the best results in the subsequent analysis and drawing.

3.2.1 Output sequence names

[saaps -i your/seq/path -osn -o your/output/path]

Code explanation: -i your/seq/path indicates that the matched sequence file is passed. -osn indicates the sequence name in the output sequence file. By default, the output is in a .csv table. -o your/output/path indicates the output path. If this parameter is not specified, the result will be output in the default path.After the program is executed, a file named SeqName.csv will be generated, which can be used for users to modify the sequence names individually. It is recommended to save the modified new names in the second column, so that subsequent programs can modify the sequence names in batches.

3.2.2 Change sequence names

[saaps -i your/seq/path -csn your/names/file/path -o your/output/path]

Code explanation: In this step, -csn your/names/file/path represents the matching table of the old and new names in the sequence, which is the table output in the previous step. The modified sequence file is named "NewNameSeq.txt" by default.

3.3 Polymorphism analysis

Although SAAPS was developed only for the analysis of SAP, the basis of polymorphism analysis is statistical analysis of the symbols and frequencies of each site in the sequence, so SAAPS can also be applied to sequences such as nucleotides.

[saaps -i your/seq/path -cp -pp -o your/output/path]

Code explanation: -csn your/names/file/path represents the matching table of the old

and new names in the sequence, which is the table output in the previous step. -cp represents to activate the computational polymorphism function. -pp represents that the polymorphism result is plotted.

The statistical result of polymorphism analysis will generate three files named [Concise_Result.txt], [Detail_Result.txt], and [Seq_Matrix.csv]. If the drawing parameter (-pp) is set, A resulting graph (default) named [SAPs.pdf] is generated accordingly. The main result files are explained as follows:

Concise_Result.txt: a concise result of polymorphism analysis, which mainly records the frequency of amino acids at each site, and is also an input file for the subsequent calculation of information entropy at each point.

Detail_Result.txt: detailed result of polymorphism analysis, which records the sequence name of each amino acid at each site.

Seq_Matrix.csv: indicates the sequence amino acid matrix.

SAPs.pdf: Polymorphism distribution map.

3.4 Shannon entropy calculation

In the SAAPS calculation results, information content (IC value) is used to replace information entropy, and the higher the IC value, the more conservative the amino acid at the site. During SAAPS information entropy analysis, two result files, [Shannon_log.txt] and [Shannon_IC_Result.csv,] are generated by default.

[saaps -i your/Concise/result/path -sha -pic -o your/output/path]

Code explanation: When calculating information entropy, we need to pass the Concise_Result.txt file in the previous step of polymorphism analysis, -sha means to activate the information entropy calculation function, -pic means to draw the information entropy result, and a picture file named IC-Plot.pdf is generated in the output path by default.

Shannon_log.txt: indicates the simple statistical result of IC analysis.

Shannon_IC_Result.csv: IC values of all sites in sequence. The table contains all site IC values, which are convenient for the subsequent drawing of IC figure.

IC-Plot.pdf: Sequence IC scatter figure.

3.5 Polymorphism site screening and Onehot encoding

[saaps -i your/Seq/Matrix/path -sr your/IC/Result/path -smin ICmin -smax ICmax -o your/output/path]

Code explanation: During the Onehot encoding process, the [Seq_Matrix.csv] file from the polymorphism analysis in the previous step and the [Shannon_IC_Result.csv] file from the information entropy calculation need to be passed in. If only the sites within the threshold range need to be converted, You can use [-smin] and [-smax] to set the IC threshold range. In the Onehot encoding step, the software automatically generates 4 files: [OneHot-Original.csv], [One-Hot-ConciseMatrix.csv], [OneHot-Transform.csv], and [One-Hot-ForIntersect.csv].

OneHot-Original.csv: a table of polymorphic loci selected according to the IC threshold range and used for onehot encoding.

One-Hot-ConciseMatrix.csv: the process file after the Onehot encoding of [OneHot-Original.csv], where each amino acid is represented by the corresponding binary number.

OneHot-Transform.csv: the result of upgrading the dimensions according to the [One-Hot-ConciseMatrix.csv file], in which the binary list of each point is expanded into the corresponding number dimensions, and each dimension only contains the corresponding values in the binary list.

One-Hot-ForIntersect.csv: indicates the record list of sequence polymorphism loci. The table records the amino acids of each sequence at the polymorphic site, which can be used for differential complement analysis of subsequent sets, etc.

3.6 Dimensionality reduction and clustering

The function of Onehot encoding is to convert classified data into numerical data that can be quantitatively analyzed. After the onehot encoding conversion, the information of each location of each sequence is converted into high-dimensional binary data. Therefore, in order to better analyze the sequence features, we usually adopt a certain dimensionality reduction algorithm to analyze it and observe the relationship between each sequence. Here we take the most commonly used PCA algorithm as an example.

[saaps -i your/Transform/result/path -pca -pc -o your/output/path]

Code explanation: In the process of dimensionality reduction analysis, the [OneHot-Transform.csv] file in the previous step needs to be passed in; [-pca] indicates that the PCA dimensionality reduction function is activated. [-pc] means to draw a scatter plot based on the dimensionality reduction results. This process produces two result files called [PCA.csv] and [PCA-Plot.pdf].

PCA.csv: PCA calculation result of sequence file.

PCA-plot.pdf: Based on the PCA analysis results, the scatter Plot is generated according

to the default parameters.
