# Supplementary material for: Information-Entropy-Based Single Amino Acid Polymorphism Analysis Reveals Functional Variance of Enterovirus 2A Proteases
Source: Microorganisms. 2026 Jul 24;14(8):1616. doi: 10.3390/microorganisms14081616 (PMC13515151; doi:10.3390/microorganisms14081616)
Supplement: Supplementary file 1 [file microorganisms-14-01616-s001.zip › SupplementTables.pdf]

Supplementary Table S1: All Virus Sequences Used in the Study

Virus	Accession Number	Known hosts*
CVA2	AY421760	*Homo sapiens*
CVA3	AY421761	*Homo sapiens*
CVA4	AY421762	*Homo sapiens*
CVA5	AY421763	*Homo sapiens*
CVA6	AY421764	*Homo sapiens*
CVA7	AY421765	*Homo sapiens*
CVA8	AY421766	*Homo sapiens*
CVA10	AY421767	*Homo sapiens*
CVA12	AY421768	*Homo sapiens*
CVA14	AY421769	*Homo sapiens*
CVA16	U05876	*Homo sapiens*
EV-A71	U22521	*Homo sapiens*
EV-A76	AY697458	*Homo sapiens*
EV-A89	AY697459	*Homo sapiens*
EV-A90	AY697460	*Homo sapiens*
EV-A91	AY697461	*Homo sapiens*
EV-A92	EF667344	*Macaca mulatta; Macaca nemestrina; Cercocebus atys*
EV-A114	KU355876	*Homo sapiens*
EV-A119	ON809571	*Homo sapiens; Gorilla gorilla; Pan troglodytes*
EV-A120	LK021688	*Homo sapiens*
EV-A121	KU355877	*Homo sapiens*
EV-A122	AF326754	*Macaca fascicularis; Macaca mulatta; Macaca nemestrina*
EV-A123	AF326761	*Macaca fascicularis*
EV-A124	AF326764	*Macaca mulatta; Macaca nemestrina*
EV-A125	AF326750	*Papio cynocephalus*

Note: This table is adapted from the Picornaviridae database (https://picornaviridae.com/ensavirinae/enterovirus/ev-a/ev-a.htm), all sequences used in this study correspond to prototype strains for each EV-A serotype.

*, *Gorilla gorilla*, gorilla; *Pan troglodytes*, chimpanzee; *Homo sapiens*, man; *Macaca mulatta*, rhesus macaque; *Macaca nemestrina*, pigtail macaque; *Macaca fascicularis,* long tailed or crab eating macaque*;* *Cercocebus atys*, sooty mangabey; *Papio cynocephalus*, yellow baboon.

Supplementary Table S2: Results of EV-A 2A Protease Protest Detection

Model	deltaBIC	BIC	BICw	-lnL
LG+G	0.00	3827.26	0.79	1793.38
WAG+G	3.55	3830.81	0.13	1795.15
LG+I+G	4.84	3832.10	0.07	1793.29
WAG+I+G	8.52	3835.78	0.01	1795.13
CpREV+G	18.78	3846.04	0.00	1802.77
CpREV+I+G	23.79	3851.05	0.00	1802.77
RtREV+G	30.84	3858.10	0.00	1808.79
VT+G	31.09	3858.35	0.00	1808.92
JTT+G	33.02	3860.28	0.00	1809.88
RtREV+I+G	35.66	3862.92	0.00	1808.70
VT+I+G	35.97	3863.23	0.00	1808.86
Dayhoff+G	37.42	3864.68	0.00	1812.08
JTT+I+G	37.71	3864.97	0.00	1809.72
DCMut+G	37.71	3864.97	0.00	1812.23
Dayhoff+I+G	42.16	3869.42	0.00	1811.95
DCMut+I+G	42.45	3869.71	0.00	1812.09
Blosum62+G	42.79	3870.05	0.00	1814.77
Blosum62+I+G	47.80	3875.06	0.00	1814.77
WAG+I	50.01	3877.27	0.00	1818.38
FLU+G	50.19	3877.45	0.00	1818.47
FLU+I+G	54.84	3882.10	0.00	1818.29
LG+I	57.40	3884.66	0.00	1822.07
VT+I	79.75	3907.01	0.00	1833.25
CpREV+I	80.75	3908.01	0.00	1833.75
Dayhoff+I	84.22	3911.49	0.00	1835.49
DCMut+I	84.23	3911.49	0.00	1835.49
RtREV+I	85.11	3912.37	0.00	1835.93
JTT+I	87.37	3914.63	0.00	1837.06
HIVb+G	93.32	3920.58	0.00	1840.04
HIVb+I+G	97.24	3924.51	0.00	1839.49
Blosum62+I	97.76	3925.02	0.00	1842.26
WAG	116.83	3944.10	0.00	1854.30
FLU+I	122.69	3949.95	0.00	1854.72
LG	128.94	3956.20	0.00	1860.35
CpREV	141.42	3968.68	0.00	1866.59

VT	148.94	3976.21	0.00	1870.35
DCMut	156.63	3983.89	0.00	1874.20
Dayhoff	156.66	3983.92	0.00	1874.21
RtREV	159.14	3986.40	0.00	1875.45
Blosum62	161.90	3989.16	0.00	1876.83
JTT	162.24	3989.50	0.00	1877.00
HIVb+I	169.06	3996.32	0.00	1877.90
FLU	199.63	4026.89	0.00	1895.69
HIVw+G	204.56	4031.82	0.00	1895.66
HIVw+I+G	208.71	4035.97	0.00	1895.22
MtREV+G	212.83	4040.09	0.00	1899.79
MtREV+I+G	217.84	4045.10	0.00	1899.79
MtArt+G	222.11	4049.37	0.00	1904.43
MtArt+I+G	227.08	4054.34	0.00	1904.41
HIVb	263.10	4090.36	0.00	1927.43
HIVw+I	282.96	4110.22	0.00	1934.85
MtMam+G	297.46	4124.72	0.00	1942.10
MtMam+I+G	302.47	4129.73	0.00	1942.11
MtREV+I	310.25	4137.51	0.00	1948.50
MtREV	364.15	4191.41	0.00	1977.95
HIVw	392.58	4219.84	0.00	1992.17
MtArt+I	393.14	4220.40	0.00	1989.95
MtArt	471.69	4298.95	0.00	2031.73
MtMam+I	501.26	4328.52	0.00	2044.01
MtMam	580.71	4407.97	0.00	2086.24

Note: The "BIC value" is a criterion used to evaluate the quality of a model, which can balance the goodness - of - fit and complexity of the model. "deltaBIC" represents the difference in BIC between the current model and the optimal model; "BICw" is the model weight corresponding to the BIC value, which indicates the relative probability of a certain model being the best model among all candidate models; "-lnL" is the negative value of the log - likelihood of the model. The log - likelihood measures the goodness - of - fit of the model to the data. A larger log - likelihood value means a better fit of the model to the data.

Supplementary Table S3：Results of EV-A 2A Protease MEGA Detection

**Model**	**BIC**	**AICc**	**lnL**
LG+G	3974.943176	3677.19765	-1789.963321
WAG+G	3979.313185	3681.567659	-1792.148325
LG+G+I	3982.731823	3678.810103	-1789.742889
WAG+G+I	3985.647392	3681.725671	-1791.200673
JTT+G	4005.034498	3707.288972	-1805.008982
rtREV+G	4005.5933	3707.847774	-1805.288383
cpREV+G	4005.630216	3707.88469	-1805.306841

Dayhoff+G	4011.567931	3713.822406	-1808.275699
rtREV+G+I	4011.721506	3707.799785	-1804.237731
JTT+G+I	4013.182547	3709.260826	-1804.968251
cpREV+G+I	4013.874496	3709.952776	-1805.314226
Dayhoff+G+I	4021.857571	3717.935851	-1809.305763
WAG+I	4023.486594	3725.741068	-1814.23503
LG+I	4032.005354	3734.259828	-1818.49441
rtREV+I	4057.729509	3759.983983	-1831.356488
JTT+I	4058.894425	3761.148899	-1831.938946
Dayhoff+I	4061.063815	3763.318289	-1833.023641
cpREV+I	4067.555995	3769.810469	-1836.269731
WAG	4089.334739	3797.766517	-1851.273858
LG	4097.166917	3805.598695	-1855.189947
cpREV	4127.88203	3836.313809	-1870.547504
JTT	4129.100193	3837.531971	-1871.156585
Dayhoff	4129.402416	3837.834194	-1871.307697
rtREV	4129.924032	3838.35581	-1871.568505
WAG+G+F	4145.282039	3730.379536	-1796.952397
LG+G+F	4149.861128	3734.958625	-1799.241941
WAG+G+I+F	4153.599697	3732.542248	-1796.996471
LG+G+I+F	4157.989344	3736.931896	-1799.191294
cpREV+G+F	4170.400405	3755.497902	-1809.51158
Dayhoff+G+F	4171.717595	3756.815092	-1810.170175
rtREV+G+F	4172.313175	3757.410672	-1810.467965
cpREV+G+I+F	4178.746283	3757.688834	-1809.569763
Dayhoff+G+I+F	4179.542237	3758.484788	-1809.967741
rtREV+G+I+F	4179.775685	3758.718236	-1810.084464
JTT+G+F	4182.724731	3767.822228	-1815.673743
JTT+G+I+F	4190.776851	3769.719402	-1815.585048
mtREV24+G	4191.315182	3893.569656	-1898.149324
WAG+I+F	4193.025263	3778.12276	-1820.824009
mtREV24+G+I	4199.275446	3895.353726	-1898.014701
mtREV24+G+F	4202.942007	3788.039504	-1825.782381
LG+I+F	4205.027092	3790.124589	-1826.824923
mtREV24+G+I+F	4210.864229	3789.80678	-1825.628736
Dayhoff+I+F	4216.307572	3801.405069	-1832.465164
rtREV+I+F	4225.508769	3810.606266	-1837.065762
cpREV+I+F	4225.654033	3810.75153	-1837.138394
JTT+I+F	4235.156093	3820.25359	-1841.889424
WAG+F	4255.331234	3846.584803	-1856.09175
mtREV24+I+F	4262.314641	3847.412138	-1855.468698
LG+F	4272.841125	3864.094695	-1864.846696
Dayhoff+F	4285.224431	3876.478001	-1871.038349

cpREV+F	4287.349302	3878.602871	-1872.100784
mtREV24+I	4288.436179	3990.690653	-1946.709823
rtREV+F	4295.311789	3886.565359	-1876.082028
JTT+F	4305.917174	3897.170744	-1881.38472
mtREV24+F	4329.149389	3920.402958	-1893.000827
mtREV24	4338.327985	4046.759763	-1975.770481

Note: The "BIC value" is a criterion used to evaluate the quality of a model and can balance the goodness-of-fit and complexity of the model. AICc is a modified version of the Akaike Information Criterion, specifically designed for situations with small sample sizes to avoid over-penalizing the complexity of the model. To avoid overfitting when the sample size is small, a penalty term is added to AIC to form AICc. AICc is applicable to small sample data, especially when the number of samples is close to the number of parameters. The smaller its value is, the better the model is. "lnL" is the log-likelihood value of the model, indicating how well the model fits the data.

Supplementary Table S4：Results of IC values for all sites of 2A protease.

SITE	IC
Site 1	4.32193
Site 2	2.54905
Site 3	4.07964
Site 4	4.32193
Site 5	4.32193
Site 6	4.32193
Site 7	4.32193
Site 8	4.32193
Site 9	4.07964
Site 10	3.35098
Site 11	4.32193
Site 12	4.07964
Site 13	3.55781
Site 14	3.79257
Site 15	3.6
Site 16	3.79257
Site 17	3.33234
Site 18	3.83975
Site 19	4.32193
Site 20	4.07964
Site 21	4.32193
Site 22	4.32193
Site 23	4.32193
Site 24	4.07964
Site 25	2.29019

Site 26	2.5324
Site 27	4.32193
Site 28	4.07964
Site 29	1.9348
Site 30	4.07964
Site 31	4.07964
Site 32	3.68237
Site 33	4.32193
Site 34	4.07964
Site 35	3.45561
Site 36	3.13896
Site 37	1.91964
Site 38	4.32193
Site 39	4.32193
Site 40	3.83975
Site 41	4.07964
Site 42	3.91975
Site 43	3.91975
Site 44	2.85755
Site 45	3.91975
Site 46	3.17669
Site 47	4.07964
Site 48	3.30081
Site 49	4.32193
Site 50	4.07964
Site 51	4.32193
Site 52	3.13561
Site 53	4.07964
Site 54	4.32193
Site 55	4.32193
Site 56	4.32193
Site 57	2.4023
Site 58	4.32193
Site 59	2.4826
Site 60	3.0665
Site 61	4.32193
Site 62	4.32193
Site 63	4.32193
Site 64	3.41755
Site 65	4.32193
Site 66	2.98221
Site 67	4.32193
Site 68	2.49289
Site 69	3.00193

Site 70	4.07964
Site 71	4.32193
Site 72	4.32193
Site 73	4.32193
Site 74	4.07964
Site 75	3.60237
Site 76	4.32193
Site 77	2.99846
Site 78	2.99846
Site 79	4.32193
Site 80	2.96039
Site 81	3.2248
Site 82	3.02413
Site 83	2.64659
Site 84	4.07964
Site 85	2.54477
Site 86	3.55781
Site 87	3.35098
Site 88	3.83975
Site 89	3.68762
Site 90	4.32193
Site 91	4.32193
Site 92	3.00193
Site 93	4.32193
Site 94	4.32193
Site 95	4.32193
Site 96	3.91975
Site 97	4.32193
Site 98	3.33234
Site 99	2.96039
Site 100	3.79257
Site 101	3.28689
Site 102	1.67704
Site 103	4.32193
Site 104	2.78246
Site 105	3.46648
Site 106	2.85755
Site 107	3.79257
Site 108	4.32193
Site 109	4.32193
Site 110	4.32193
Site 111	4.32193
Site 112	4.32193
Site 113	4.32193

Site 114	4.32193
Site 115	4.32193
Site 116	4.32193
Site 117	3.55781
Site 118	4.32193
Site 119	4.32193
Site 120	4.32193
Site 121	3.35098
Site 122	4.32193
Site 123	3.46648
Site 124	4.07964
Site 125	3.37924
Site 126	2.98221
Site 127	4.32193
Site 128	4.32193
Site 129	2.78645
Site 130	3.68762
Site 131	3.91975
Site 132	4.32193
Site 133	4.07964
Site 134	4.32193
Site 135	4.32193
Site 136	4.07964
Site 137	2.89497
Site 138	4.32193
Site 139	4.32193
Site 140	4.07964
Site 141	3.91975
Site 142	4.32193
Site 143	4.07964
Site 144	3.68762
Site 145	3.33234
Site 146	3.79257
Site 147	3.41755
Site 148	4.32193
Site 149	4.32193
Site 150	4.32193

Supplementary Table S5: The Number of Common DEGs in Each Group.

Class	Common DEGs
Group1	44
Group3	32

Group2	7
Group2（with EV-A124 excluded）	7

Supplementary Table S6: Changes in common DEGs among EV-A92, EV-A122, EV-A123 and EV-A124

Viruses	Common DEGs	IC range
EV-A92、EV-A122、EV-A123	30	1.677~4.322
EV-A92、EV-A122、EV-A123	30	1.761~4.022
EV-A124、EV-A92、EV-A122、EV-A123	28	2.735~4.022
EV-A92、EV-A122、EV-A123	30	3.022~4.022
EV-A92、EV-A122、EV-A123	30	3.274~4.022
EV-A92、EV-A122、EV-A123	30	3.274~4.322
